# Adherence to MIND Diet and Risk of Recurrent Depressive Symptoms: Prospective Whitehall II Cohort Study

**DOI:** 10.3390/nu16234062

**Published:** 2024-11-26

**Authors:** Husnain Arshad, Daisy Recchia, Jenny Head, Kathleen Holton, Joanna Norton, Mika Kivimaki, Tasnime N. Akbaraly

**Affiliations:** 1 INSERM (Institut National de Santé et de Recherche Médicale), UVSQ (Université de Versailles Saint-Quentin-en-Yveline), Paris-Saclay Université, CESP (Centre de Recherche en Epidémiologie et Santé des Populations), F-75018 Paris, France; drhusnain@live.com; 2 INSERM (Institut National de Santé et de Recherche Médicale), MMDN (Mécanismes Moléculaires des Démences Neurodégénératives), Univ Montpellier, F-34095 Montpellier, France; daisy.recchia.pro@gmail.com; 3 Department of Epidemiology and Public Health, University College London, London WC1E 6BT, UK; j.head@ucl.ac.uk; 4 Departments of Health Studies and Neuroscience, American University, Washington, DC 20016, USA; holton@american.edu; 5 Institute for Neurosciences of Montpellier (INM), INSERM (Institut National de Santé et de Recherche Médicale), University of Montpellier, F-34295 Montpellier cedex 5, France; joanna.norton@inserm.fr; 6 Brain Sciences, University College London, London WC1E 6BT, UK; m.kivimaki@ucl.ac.uk; 7 Desbrest Institute of Epidemiology and Public Health (IDESP), INSERM (Institut National de Santé et de Recherche Médicale), Univ Montpellier, F-34398 Montpellier, France

**Keywords:** nutritional epidemiology, depressive disorders, prospective cohort study

## Abstract

Background/Objectives: This study examined the association between adherence to the Mediterranean dietary approaches to stop hypertension Intervention for Neurodegenerative Delay (MIND) diet, its components, and recurrent depressive symptoms (DepSs). Methods: The analyses included 4824 participants (73% men, mean age = 61; SD = 5.9) from the British Whitehall II cohort study. The MIND diet scores were derived from a validated 127-item food frequency questionnaire in 2002–2004. DepSs were assessed by the Center for Epidemiologic Studies Depression Scale (score ≥ 16) or by use of antidepressant drugs, and recurrence was defined as having DepSs in at least two of the four repeated measurements in the 2002–2004, 2007–2009, 2012–2013, and 2015–2016 follow-up phases. Results: Recurrent DepSs were observed in 13.3% of the participants over 13 years of follow-up. After adjusting for potential confounders, participants in the highest tertile of the MIND diet score (where a higher score represents a higher diet quality) had 26% lower odds of experiencing recurrent DepSs (OR = 0.74; 95% CI 0.58–0.93) compared to those in the lowest tertile. In mutually adjusted analyses of 14 MIND diet components in relation to recurrent DepSs, independent associations were observed for green leafy vegetables (OR = 0.59, 95% CI: 0.45–0.78), other vegetables (OR = 0.43, 95% CI:0.24–0.77), and berries (OR = 0.74, 95% CI: 0.61–0.89). Conclusions: In this British prospective cohort, good adherence to the MIND diet, particularly to the recommendations for vegetables and berries, was associated with a lower risk of recurrent depressive symptoms, independent of socio-economic, health behavior, and health status factors, including baseline cognitive impairment and antecedents of DepSs.

## 1. Introduction

The field of nutritional psychiatry has evolved rapidly during the last decade, with a growing amount of evidence suggesting an association between overall diet quality and depression. A meta-analysis of prospective cohort studies found that adherence to healthier dietary behaviors reduces the risk of depression by approximately 30% [1]. Accordingly, randomized controlled trials also suggest a significant reduction in depressive symptoms (DepSs) in clinical samples through whole-diet interventions [2]. However, clinical trials testing multi-nutrient supplementation have not observed benefits in intervention groups relative to placebo [3,4,5].

These findings may reflect the difficulty in capturing the effects of long-term diet modification in interventional studies, especially in non-clinical populations, and, thus, reaffirm the importance of carrying out analyses in observational prospective cohorts to better understand the associations between adherence to dietary recommendation and depression trajectories in the general population. On another note, while several diet quality indices have been shown to be associated with depression, none of them have been specifically designed for depressive disorders. Given the heterogeneity underlying dietary exposure, providing clear and accurate dietary recommendations to prevent depression remains a cornerstone of further progress in the field of nutritional psychiatry.

One decade ago, a new diet quality index was proposed by Morris et al. to predict age-related cognitive outcomes [6]. This index was built by combining elements from the two following well-known dietary indices: the Mediterranean diet and the Dietary Approaches to Stop Hypertension (DASH), which have been both consistently associated with risk reductions in cognitive decline, dementia, and depression. This new dietary index, called MIND, stands for “Mediterranean-DASH Intervention for Neurodegenerative Delay”. Observational studies have reported an association between adherence to the MIND diet score with slower cognitive decline [6], as well as a reduced risk of Alzheimer’s disease [7]. Recent evidence suggested that the potential neuroprotective effects of the MIND diet were superior to those of the DASH diet or the Mediterranean diet [8,9]. The MIND diet, by combining specific components of both the Mediterranean and DASH diets, may more accurately account for the dietary components important for optimal brain health. For example, the Mediterranean diet fails to capture a few essential elements of the Western diet, like processed foods and beverages that are consumed with a high frequency in non-Mediterranean Western countries [10] and have been recently reported to increase the risk of depression [11]. The MIND diet additionally focuses on the intake of specific vegetables and fruits (green vegetables and berries) rich in several nutrients and non-essential chemical compounds, whose functional properties may afford protection for cognitive decline [12,13]. While no dietary indices dedicated to predict depression exist, given the link between age-related cognitive impairment, DepSs, and depressive disorders [14], we hypothesized that a good adherence to the MIND diet might be associated with a reduced risk of recurrence of depressive symptoms at the pre-elderly/elderly stages. To date, only two studies have prospectively investigated the MIND diet–depressive outcomes association, with discordant findings. The Seguimiento University of Navarra study, which involved a large cohort of younger Spanish adults, found no significant association [15], while a cohort of older institutionalized United States participants reported a significant reduction in depression risk with a better adherence to the MIND diet [16].

Thus, we aimed, in the present study, to assess the prospective association of adherence to the MIND diet and its components with long-term recurrent DepSs over 13 years of follow-up in a well-characterized British cohort of middle-aged and elderly men and women—the Whitehall II study.

## 2. Materials and Methods

### 2.1. Study Population

Whitehall II is an ongoing cohort of British civil servants. At phase 1 (1985/88), all persons aged from 35 to 55 years working in 20 London-based departments were invited to participate by letter, and 73% agreed. The participants (N = 10,308, 6895 men and 3414 women) were self-administered a health questionnaire, and a clinical examination took place [17]. Since then, a follow-up clinical examination took place approximately every three to five years, including phase 3 (1991/94, N = 8815), phase 5 (1997/99, N = 7870), phase 7 (2002/04, N = 6967), phase 9 (2007/09, N = 6761), phase 11 (2012/13, N = 6308), and phase 12 (2015/16, N = 5632). Written consent was obtained from all the participants after a thorough explanation of the study, and research ethics approvals were renewed at each contact; the most recent approval was from the University College London Hospital Committee on the Ethics of Human Research (reference number 85/0938).

Of the 6967 participants who attended phase 7 (the baseline for the present study), we included 4824 participants who completed dietary assessments at phase 7 (2002/04) and who had data on covariates at phase 7, as well as measures of DepSs for at least two follow-ups out of four phases (2002/04, 2007/09, 2012/13, and 2015/16), as detailed in the study design and the flow-chart diagram (Supplementary Material Figure S1).

### 2.2. Assessment of Dietary Exposure

At phase 7, a validated food frequency questionnaire (FFQ) containing 127 food items was administered to assess dietary intake [18]. Details regarding the FFQ and nutrients intakes calculations have been detailed elsewhere [18].

From the FFQ, we derived the “Mediterranean-DASH Diet Intervention for Neurodegenerative Delay” (MIND) diet score. It was originally based on 15 components, 9 components considered as brain healthy (green leafy vegetables, other vegetables, nuts, berries, beans, whole-grains, fish, poultry, and olive oil) and 5 components considered as unhealthy (red meats, butter/margarine, cheese, pastries and sweets, and fried/fast food). The last component consisted of wine consumption, where only a moderate consumption was considered to be optimal. Each MIND diet component for each participant was scored as 0, 0.5, or 1 (Supplementary Table S1), and the total MIND diet score was calculated by summing the scores of each component [6]. The FFQ administered in Whitehall II did not include a question on olive oil, which was, thus, not included in the calculation of the MIND score, meaning that the highest possible score representing the healthiest diet was 14. The foods items used to build each component are detailed in Table 1. The scoring criteria for the MIND diet, along with the mean total score of the participants and the mean scores for each component, are detailed in Supplementary Table S1.

As the FFQ was also administered at phase 3 (1991/94) and phase 5 (1997/99), similar procedures were applied to compute the MIND diet score at these two phases. When dietary exposure prior to phase 7 was available, the cumulative average of the MIND diet score was calculated using the repeated measures of the MIND diet score at phase 3 (N = 4594), phase 5 (N = 3798), and phase 7 (N = 4824), allowing for assessing long-term dietary exposure (11-year exposure period) in a secondary analysis and for accounting for the possibility of measurement error. The mean scores of the cumulative averages of the MIND diet and its components are detailed in Supplementary Table S1.

### 2.3. Assessment of Recurrent Depressive Symptoms

The widely used Center for Epidemiologic Studies Depression Scale (CES-D) [19] was used to assess DepSs in all Whitehall participants, as detailed in a previous publication [20]. Briefly, this self-report scale measure is composed of 20 items matching DepSs. The participants’ frequency of each DepS item over the past week was evaluated using a four-point scale, ranging from ‘less than once a week’ to ‘5 to 7 days a week’. DepS cases were defined as participants with a CES-D score of ≥ 16 or those who were treated with antidepressants. Current medication use (generic name, brand name, or both) was self-reported by participants and was subsequently coded using the British National Formulary to determine antidepressant use. Given the varying nature of the self-reported DepSs, as measured by the CES-D [21], the recurrence of DepSs over the period from 2002/04 to 2015/16 may better reflect long-term DepSs than relying on a single measure. The recurrence of DepSs was defined as presenting DepSs at two, three, or all the four phases of follow-up (2002/04, 2007/09, 2012/13, and 2015/16), while non-recurrent cases were defined as participants reporting one or no DepS episode over the 13 years of follow-up.

### 2.4. Assessment of Covariates at Phase 7

Socio-demographic covariates included sex, age (continuous), marital status (married/cohabiting or single/divorced/widowed), education level (<secondary school, secondary school, or university level), and socio-economic status based on occupational grade (low: clerical or support, intermediate: professional/executive, and high: administrative) [17].

Behavioral covariates included total energy intake (estimated from FFQ in kCal), smoking status (ex or non-smokers and current smokers), and physical activity categorized as inactive (<1 h per week of moderate physical activity and <1 h per week of vigorous physical activity), moderately active (if neither active nor inactive), and active (>2.5 h per week of moderate physical activity or >1 h per week of vigorous physical activity) [22]. Alcohol consumption was classified in the following three categories: abstinence (no alcohol consumption in the previous week), moderate consumption (1–14 units/week in women and 1–21 units/week in men), and heavy drinkers (>14 units in women and >21 units in men).

Health-related factors consisted of a measure of body mass index in kg/m^2^, prevalent coronary heart disease assessed by clinically verified non-fatal myocardial infarction or definite angina), three cardiometabolic disorders (including hypertension (systolic/diastolic blood pressure ≥ 140/90 mm Hg, respectively, or the use of antihypertensive drugs), type 2 diabetes (fasting glucose of ≥7.0 mmol/L or 2-h post-load glucose ≥11.1 mmol/L or reported doctor diagnosed diabetes or use of diabetic medication), and dyslipidemia (triglycerides > 1.7 mmol/L or reported use of lipid-lowering drugs)), and a measure of cognitive impairment using the Mini Mental State Examination (score ≤ 27) Details about the collection of these data are provided elsewhere [21].

Antecedents of DepSs were also considered as covariates. As the CES-D was not administered in the Whitehall II study prior to phase 7 (2002/04), we used the four-item depression subscale from the general health questionnaire (GHQ) (with a cut-off point of ≥4 out of 12 defining GHQ depression) [20]. Participants with GHQ depression at phase 3 (1991/94), phase 5 (1997/99), or phase 7 (2002/04) or those treated by antidepressants at phase 3 (1991/94) or phase 5 (1997/99) were defined as having antecedents of DepSs.

All health-related factors added to the statistical models were used as binary categorical variables (yes/no), except for the body mass index (kg/m^2^), which was analyzed as a continuous variable. All these covariates, with the exception of cognitive impairment, were also assessed in phase 3 (1991/94) and included in the sensitivity analyses.

### 2.5. Statistical Analyses

Participants who were excluded were compared to those who ended up being included in the analyses to evaluate whether or not any biases were present. The participant characteristics for those who were included in analyses were also compared according to the presence/absence of recurrent DepSs over the years and according to the tertiles of their MIND diet scores. These comparisons were performed using Chi-square tests (for categorical variables), ANOVA, or Student *t* tests (for continuous variables) as appropriate.

To test the potential interaction between covariates and the MIND diet in regard to recurrent DepSs, bivariate analyses were conducted. No interactions were observed between covariates and dietary exposure in regard to DepS outcomes.

Associations between the MIND diet scores (analyzed as tertiles) assessed in 2002/04 and recurrent DepSs assessed at four different time points over 13 years of follow-up (2002/04–2015/16) were examined using logistic regression models. The models were first adjusted for age, sex, and energy intake (Model 1). Additional adjustments were made for ethnicity, marital status, education level, socio-economic status, and health behaviors, including smoking status, physical activity, and alcohol consumption (Model 2), as well as for health-related factors including coronary heart diseases, hypertension, type 2 diabetes, dyslipidemia, body mass index, cognitive impairment, and antecedents of DepSs (Model 3). The tests of linear trends across categories were assessed by considering the tertiles of the MIND diet scores as ordinal variables (with the lowest tertile as a reference).

A set of supplementary analyses were conducted as sensitivity analyses. First, logistic regression models were applied to estimate the association between the cumulative average of the MIND diet score assessed over 11 years of exposure (from phase 3 to phase 7) and recurrent DepSs. Models were adjusted for sociodemographic (sex, age, cumulative average of total energy intakes, ethnicity, SES, marital status, and education level), health behavior (smoking status and physical activity), and health status factors (cardio-metabolic factors) and antecedents of depression assessed at phase 3 (1991/94). Second, as the MIND diet was originally derived to predict cognitive health, in order to isolate the association between the MIND diet and recurrent DepSs independent of cognitive health, we repeated the main analyses by excluding the participants with cognitive impairment at baseline. Third, to further assess the directionality of the association between the MIND diet and the recurrence of DepSs, the main analyses were repeated by excluding, first, the 165 participants on antidepressant treatment prior to phase 7 (N= 4659) and, second, the 609 participants with antecedents of GHQ depression at phase 5 (1997/99)—5 years before the assessment of diet exposure (N = 4215). Fourth, the main analyses were repeated by adjusting for the self-reported use of vitamins/minerals or other food supplements (yes, no) collected from the FFQ at phase 7 for 4538 participants. The association between the MIND diet and recurrence of DepSs was also estimated in supplement users and non-users.

A second set of analyses was conducted to study the associations between the score on each individual component of the MIND diet and recurrent DepSs. Logistic regression models were conducted to estimate the odds of recurrent DepSs associated with each MIND diet component (analyzed as a continuous variable) adjusted for the covariates listed above, with the exception of alcohol. To assess whether the MIND diet component–recurrent DepSs association was independent of other components, models were additionally adjusted for a modified MIND score. This modified MIND diet score was based on the total MIND diet score without the component i (modified MIND diet score = Total MIND diet score − score of the component i). All analyses were conducted using the SAS software, version 9.4 (SAS Institute, Cary, NC, USA).

## 3. Results

### 3.1. Characteristics of Population Study

Of the 6967 participants at baseline (Whitehall study phase 7), the current analyses were carried out for 4824 participants (3526 men and 1298 women). As detailed in Supplementary Material Table S2, the participants included were more likely to be men, younger, white, married, and have a higher socio-economic and education status compared to those excluded. They were also more likely to be physically active and less likely to have cardio-metabolic disorders, cognitive impairment, and recurrence of DepSs. No significant difference was observed for the mean MIND diet score between the participants included and those excluded from the current analyses.

The characteristics of the participants according to the recurrence of DepSs are presented in Supplementary Material Table S3. Over the 13 years of follow-up (2002/04–2015/16), 640 participants (13.3%) reported having recurrent DepSs. Participants with recurrent DepSs were more likely to be women, non-white, living alone (single/divorced/widowed), with a lower socioeconomic status, and have poor health behaviors. Interestingly they were more likely to report using vitamins/minerals and food supplements. Among health-related factors, the participants with recurrent DepSs were more likely to have dyslipidemia, cognitive impairment, and antecedents of DepS (based on GHQ).

The characteristics of the participants according to the tertiles of their MIND diet scores at baseline are presented in Table 2. Participants in the highest tertile of MIND diet score were more likely to be women, older in age, non-white, married or cohabiting, who had a higher education level. They were also more likely to have healthier lifestyle behaviors (ex or non-smokers and physically active) and to report the use of vitamins/minerals or food supplements. Among health-related factors, a low prevalence of dyslipidemia, DepS antecedents, and a lower body mass index were associated with a higher adherence to the MIND diet.

### 3.2. Association Between MIND Diet Scores and Recurrent DepSs

**Figure 1:**
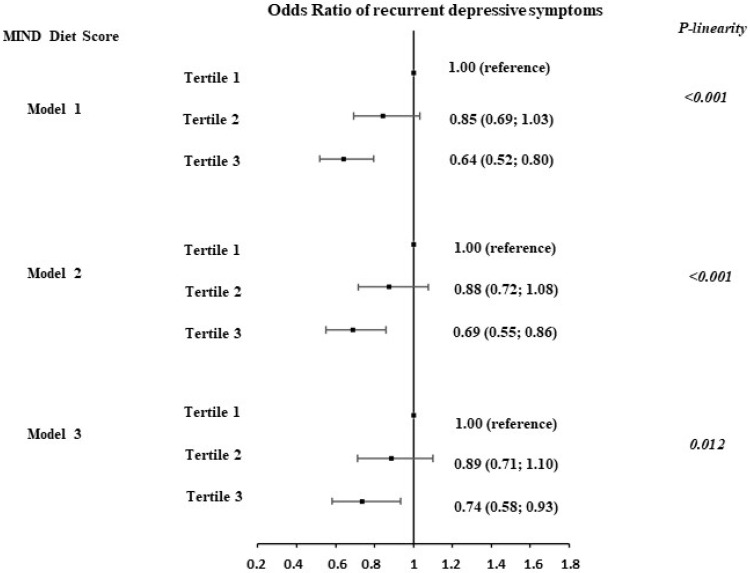
Association between MIND diet score assessed in 2002/04 and recurrence of depressive symptoms (DepSs) over 13 years of follow-up (2002/2004 to 2015/16) in 4824 Whitehall II participants (3526 men and 1298 women). Model 1 adjusted for: sex, age, and total energy intake. Model 2 adjusted for: M1 + ethnicity, marital status, socio-economic status, education level, smoking status, alcohol consumption, and physical activity. Model 3 adjusted for: M2 + coronary heart disease, hypertension, diabetes, dyslipidemia, body mass index, cognitive impairment, and antecedents of depressive symptoms.

The results of logistic regression models showed a statistically significant association between the MIND diet tertiles and recurrent DepSs assessed over 13 years of follow-up (Figure 1). After adjustment for age, sex, and total energy intake, the participants in the top tertile of the MIND diet score had lower odds of recurrent DepSs (Model 1, OR = 0.64, 95%CI: 0.52 to 0.80) compared to the participants in the lowest tertile. This association remained statistically significant after adjustments for socio-demographic variables and health behaviors (Model 2, OR = 0.69, 95%CI: 0.55 to 0.86). Further adjustments for cardio-metabolic health factors at baseline and antecedents of DepS did not substantially attenuate the results (Model 3, OR = 0.74, 95%CI: 0.58 to 0.93). In analyses considering the MIND diet tertiles as ordinal variables, a linear trend across the tertiles of the MIND diet was observed (*p* linearity = 0.012).

We conducted four sets ofe additional analyses to examine the robustness of the association. First, to represent long-term dietary intakes and reduce measurement errors, we estimated the odds of recurrent DepSs associated with the cumulative average MIND diet score with the available MIND score measures from baseline (2002/2004) and the following two preceding phases: phase 3 (1991/93) and 5 (1997/99). In models successively adjusted for socio-demographic, health behavior, and health status measures assessed at phase 3 (Supplementary Material Figure S2), higher cumulative average MIND diet scores assessed over the 11 years of exposure period were found to be associated with lower odds of recurrent DepSs, confirming the trends reported in the main analyses.

Second, to further assess whether the association was found to be independent of cognitive functioning, we conducted sensitivity analyses in which we excluded the 612 participants with cognitive impairment at baseline (2002/04), and similar results than those reported in main analyses were observed (for the top tertile of MIND diet compared to the lowest tertile: OR = 0.70, 95%CI: 0.56 to 0.89, Supplementary Material Table S4).

Third, while the main analysis showed that the MIND diet score–recurrent DepSs association was independent of antecedents of DepSs, two supplementary analyses were conducted to assess whether these associations remained after excluding (1) participants under anti-depressive drugs treatment 11 years before baseline and (2) participants with GHQ depression five years before baseline (results are shown in Supplementary Material Tables S5 and S6, respectively). The results showed that exclusion of these participants did not substantially affect the association between tertiles of the MIND diet score and odds of recurrent DepSs.

Finally, while self-reported use of vitamins/minerals and other food supplements was shown to be more prevalent in participants in the highest tertile of the MIND diet score (Table 2), we conducted a model in which the full-adjusted association between the MIND diet and recurrence of DepSs additionally accounted for the use of supplements. The results reported in Supplementary Material Table S7 show a slight intensification of the association compared to those described in main analyses. Even if no significant interaction was reported between the MIND diet and the use of supplements regarding the recurrence of DepS, our additional analyses highlighted a significant association between a good adherence to the MIND diet and lower odds of recurrent DepSs in non-supplement users (n = 2328), while no significant association was reported in participants self-reporting the use of vitamins/minerals or other food supplements (n = 2210) (Supplementary Material Table S7).

### 3.3. Association Between Independent Components of MIND Diet and Recurrent DepS

The results of the logistic model estimated the odds of recurrent DepSs for each increase of 1 point in the component score (with 1 point representing the most favorable intake versus 0 points representing the most unfavorable intake). Of the 14 components of the MIND diet, the consumption of green vegetables (OR = 0.55, 95%CI: 0.42 to 0.72), all other vegetables (OR = 0.37, 95%CI: 0.21 to 0.66), berries (OR = 0.71, 95%CI: 0.59 to 0.87), fish (not fried) (OR = 0.70, 95%CI: 0.54 to 0.91), and a low intake of fast/fried foods (OR = 0.74, 95%CI: 0.56 to 0.98) were associated with lower odds of DepS recurrence after accounting for sex, age, and total energy intake. A marginally significant association was also observed for whole grain and wine component intake (Supplementary Material Table S8).

**Figure 2:**
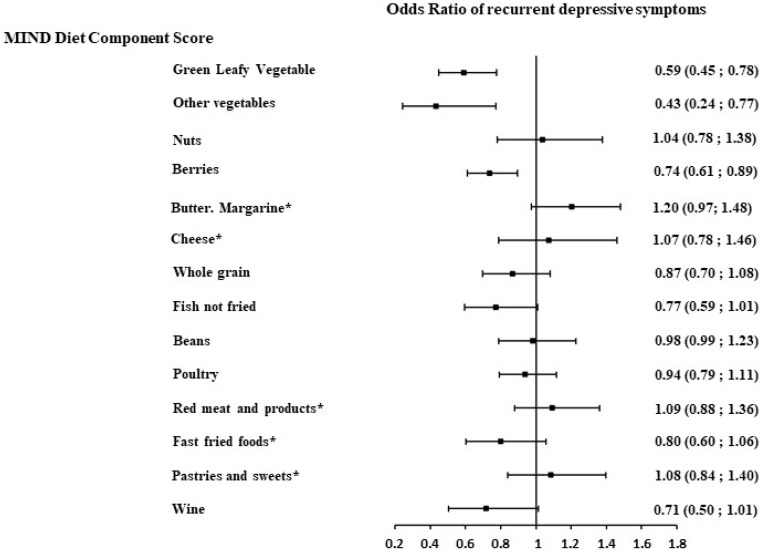
Association between each MIND diet component (2002/04) and the recurrent depressive symptoms (DepSs) over 13 years of follow-up in 4824 Whitehall II participants after taking into account other MIND diet components. Models were adjusted for sex, age, total energy intake, and modified MIND diet score (total MIND diet score excluding the component under examination). MIND diet component score: higher score corresponds to higher intakes of the component. * MIND diet component score: higher score corresponds to lower intakes of the component.

**Figure 3:**
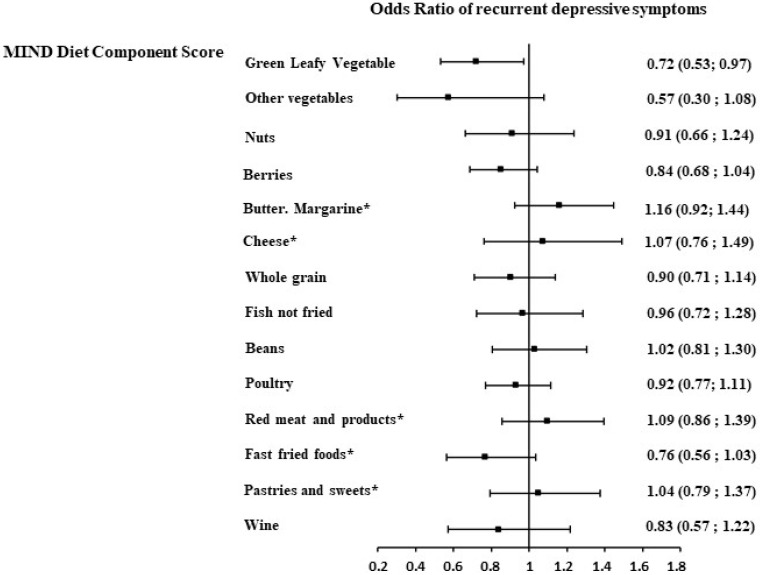
Association between each MIND diet component (2002/04) and the recurrent depressive symptoms (DepSs) over 13 years of follow-up in 4824 Whitehall II participants after taking into account other MIND diet components, socio-economic, health behavior, and health status factors. Models were adjusted for sex, age, total energy intake, modified MIND diet score (total MIND diet score excluding the component under examination), ethnicity, marital status, socio-economic status, education level, smoking status, physical activity, coronary heart diseases, hypertension, diabetes, dyslipidemia, body mass index, cognitive impairment, and antecedent of depressive symptoms. MIND diet component score: higher score corresponds to higher intakes of the component; * MIND diet component score: higher score corresponds to lower intakes of the component.

To further assess whether these MIND diet components–recurrent DepS associations were independent of other components, the regression models were additionally adjusted for a modified MIND diet score, where the component under examination was excluded from the total score. While similar trends were observed, a statistically significant association was observed for green leafy vegetables, all other vegetables, and berries (Figure 2). The fish consumption, fast fried food intake, and moderate wine intake components were found to be marginally significantly associated with recurrent DepSs. Further adjustment for other socio-economic factors, health behavior, health-related factors, including antecedents of DepSs (from GHQ), substantially attenuated these associations (Figure 3), with only the green leafy vegetable component remaining significantly associated with recurrent DepSs (OR= 0.72, 95%CI: 0.53 to 0.97) (Figure 3).

Lastly, we assessed the extent to which individual MIND diet components may explain the overall MIND diet–DepSs association. As shown in Supplementary Material Table S9, after the serial exclusion of each component, the association between each modified MIND score and DepSs remained marginally significant, suggesting that the association between the MIND diet and DepS recurrence was not driven by any individual component.

## 4. Discussion

A statistically significant association between a better adherence to the MIND diet and lower odds of recurrent depressive symptoms was observed in the current investigation carried out on 4824 British civil servants. The participants in the highest adherence tertile to the MIND diet were 26% less likely to have recurrent depressive symptoms over the 13 years of follow-up compared to participants in the lowest tertile. This association was found to be independent of socio-demographic, health behavior, and health-related factors, including prevalent cognitive impairment, antecedents of depressive symptoms at the time of diet assessment, and the use of vitamins/minerals or other food supplements.

### 4.1. Comparison of MIND Diet–DepS Relationship with Other Studies and Possible Explanation Underlying the Association

Previously, the prospective association between MIND diet adherence and depression or DepSs had been assessed in a Mediterranean cohort of Spanish university graduates [15] and an American cohort of older adults in retirement homes and senior public housing [16]. The Spanish cohort included a relatively young population (mean age = 37.5 ± 11.5) and reported no significant association between quartiles of the MIND diet scores and incidents of depression over 10 years of follow-up (Hazards Ratio: 0.88, 95%CI: 0.68 to 1.12) [15]. The American cohort, on the other hand, included 709 elderly participants (mean age = 80.4 ± 7.2) and reported that participants in the third tertile of the MIND diet score were less likely to have DepSs over 6 years of follow-up (β −0.13, 95%CI: −0.25 to −0.02). Neither of these two studies assessed the recurrence of DepSs. In the Whitehall II cohort, we observed an association between the MIND diet and recurrent DepSs in a relatively large sample of non-Mediterranean pre-elderly participants (mean age = 61.0 ± 5.9). The lack of association between the MIND diet and depression in the Spanish cohort could be related to the relatively younger age of participants, who were less likely to benefit from the cognitive benefits of the MIND diet [15].

Our analyses exploring individual components of the MIND diet associated with recurrence of DepSs allow for highlighting the weight of green leafy vegetables. Green leafy vegetables have been described as a rich source of essential nutrients, including vitamins A, C, E, and K, with very high antioxidant properties [23,24], as well as being high in folates and a decent source of magnesium. They are also high in phytochemical such as β-carotene flavonoids. All of these nutrients have been associated with physiopathological pathways leading to depression. Dietary folates, for example, are involved in one carbon metabolism (along with vitamin B12 and riboflavin), and this process is essential for the production of dopamine, norepinephrine, and serotonin—neurotransmitters implicated in depression [25]. Additionally, one carbon metabolism plays an important regulatory role in maintaining low levels of homocysteine. Homocysteine has been associated with cerebrovascular diseases [25] and excitotoxicity (the overactivation of glutamate receptors) [26], which has also been associated with depression [27]. Magnesium’s content in green leafy vegetables has also raised interest in regard to depression. Magnesium is particularly well known for its importance as an antagonist of the N-methyl-D-aspartate glutamate receptor—the human brain’s primary excitatory neurotransmitter. By playing a role in neuromodulation and neurotransmission [28], magnesium may afford protective effects against overstimulation, which leads to neuronal death in the limbic system and cerebral cortex [29], which have been described as brain regions involved in the etiopathogenesis of depression [30,31]. Green leafy vegetables’ phytochemical nutrients, like polyphenols—described to be among the most plentiful antioxidants in the human diet [32]—might also contribute to afford protection against depression by involving metabolic regulation with strong anti-inflammatory properties [33]. 

Our multivariable-adjusted analyses also showed an almost statistically significant association of high intakes of other vegetables (other than green vegetables) with recurrent DepSs. This food component may provide additional vitamins and might also exert neuroprotective effects by reducing inflammation and oxidative stress and improving neuromodulation [34]. A marginally significant association with DepSs was also reported for the intake of berries. Berries are high in specific polyphenolic flavonoids consisting of flavan-3-ols, flavanols, and anthocyanidins (the principal ones). Potential impacts on depression physiopathology include their antioxidant properties, ability to improve vascular health and gluco-regulation, and the ability to promote positive changes in the gut microbiota [35]. The beneficial effects of the MIND diet on DepSs could also result from the higher intakes of foods rich in monounsaturated and polyunsaturated fatty acids, including nuts and oily fish, as they have been shown to reduce the risk depression in several reports [36,37]. Fish consumption also allow to provide vitamin D and constitutes the best source of vitamin D3 in the diet. Vitamin D has been shown to be a key regulator of over 900 genes, including those controlling brain serotonin synthesis (by inducing expression of the tryptophan hydroxylase 2 gene (involved in serotonin synthesis) and suppressing the expression of tryptophan hydroxylase 1 [38].

The MIND diet also restricts the intake of fried foods rich in saturated fatty acids and trans fatty acids that might result in immune-modulation processes favoring pro-inflammatory states and other biological processes involved in potential mechanisms of depression [39].

Overall, the results of our analyses examining the associations between individual components of the MIND diet and recurrent DepSs support a cumulative effect of multiple nutrients brought about by the 14 food items rather than the effect of one specific component/or nutrient. This though was also supported by the fact that the MIND diet–recurrent DepSs association was found to be independent of the self-reported use of vitamins/minerals or other food supplements, and a significant association was found in non-users of supplements.

### 4.2. Strengths and Limitations

The main strengths of this study include its prospective design and relatively large sample of 4824 participants, with 640 cases of recurrent DepSs. DepSs were measured at four time points over 13 years of follow-up, providing a robust assessment of long-term recurrent DepSs. In addition, the analyses were adjusted for a large range of potential confounding factors and were found to be independent of antecedent DepS episodes (as estimated by the GHQ). Similar trends were also observed when considering long-term adherence to the MIND diet over 11 years of exposure.

This study also had a few limitations. First, we used a semi-quantitative FFQ including only 127 food items to assess dietary intakes. This FFQ did not assess the consumption of olive oil, which is one of the major components of the MIND diet. Originally composed of 15 items, we had to adapt the MIND diet score and proposed a 14-item score, which might have led to underestimating the association between the MIND diet and DepSs, given the protective effect on olive oil on brain functions and DepSs suggested in the literature [40]. Furthermore, the FFQ is open to measurement errors common to all self-reported dietary assessments. To account for this, the main analyses were replicated by considering the cumulative average of the MIND diet score calculated using the repeated measures of MIND diet scores prior phase 7, and similar trends were observed. Furthermore, even though FFQ has been considered to be less precise than other dietary measurements methods such as dietary records, it remains the most frequent tool used in large epidemiological studies; previous prospective studies assessing the MIND diet–depression association have also used the FFQ to assess dietary intakes [15,16]. Moreover, the FFQ used in the present analyses has been validated against 7-day diet diary and biomarkers of diet in the Whitehall study [18].

Another limitation relates to the use of the CES-D scale. Although CES-D is the most commonly used and validated instrument in epidemiological studies to measure DepSs, it may fail to capture the severity or chronic nature of DepSs. To overcome this limitation, we consider repeating the CES-D measures over time to create ‘recurrent DepSs’ as the outcome of interest.

A further limitation involves the possibility that the diet and depression association is bidirectional. Thus, the directionality of the association we reported between the MIND diet score and DepSs can be challenged. To address this point, we assessed, in a supplementary analysis, the association between long-term adherence to the MIND diet score (by considering cumulative average score over the 11 years of exposure period) and the increased risk of subsequent recurrent DepSs over 13 years of follow-up, and reported similar trends. In the absence of an interaction between antecedents of DepSs and MIND diet exposure in regard to the subsequent recurrence of DepSs, we also showed that the MIND diet–DepSs association was independent of antecedent of DepSs by conducting a model adjusted for DepS antecedents. Finally, a statistically significant association between the MIND diet and DepSs was also observed in sensitivity analyses excluding participants under anti-depressive treatment 10 years before baseline and after excluding participants with depression assessed through the four-item depression subscale of the GHQ five years before baseline. We believe these arguments reinforce the role of the MIND diet adherence in the development of recurrent DepSs, without excluding the reverse causation hypothesis.

Lastly, the generalizability of our findings should be addressed in future studies, as the study population consisted of an occupational cohort from a single country, with selective participant attrition during follow-up. While we acknowledge that the replication of our findings to generalize our results is definitely needed, it is unlikely that the selection of the participants in the current analyses could have contributed to an overestimation of the MIND diet–DepSs association, as the participants included were significantly more likely to have better socio-demographic, behavioral, and health characteristics, including fewer episodes of DepSs compared to those excluded, and no significant difference was observed between participants excluded and those included in regards to adherence to the MIND diet.

## 5. Conclusions

In conclusion, the results from the current study suggest that a better adherence to MIND diet was associated with reduced odds of recurrence of DepSs in a cohort of British participants. More specifically, a greater consumption of green leafy vegetables, other vegetables, and berries appeared to be associated with having less DepSs over time. Our findings support a cumulative and possibly synergistic effect of multiple nutrients brought about by the 14 food items of the MIND diet, rather than the impact of one specific component or nutrient. Further studies are required to examine the reproducibility of our findings and the underlying mechanisms to establish a consensus on the effects of the MIND diet on recurrent depressive symptoms.

## Figures and Tables

**Table 1:** Information on foods items included in each MIND diet component.

MIND Diet Components	Foods Items from the Whitehall II Study Food Frequency Questionnaire
Green leafy vegetables	spinach, green salad, broccoli, spring greens, kale, Brussels sprouts, cauliflowers
Other vegetables	carrots, tomatoes, marrow, zucchini, parsnips, turnips, swedes, leeks, mushrooms, onions, garlic, peas, coleslaw, boiled mashed, instant or jacket potatoes, vegetable soup
Berries	strawberries, raspberries
Nuts	peanuts and other nuts
Butter and margarine	butter, margarine (all types), low fat spread
Cheese	cheese and cottage cheese
Whole grains	whole grain cereals, porridge, whole meal pasta, brown rice
Fish (not fried)	oily fish (fresh or canned), white fish (fresh or frozen), shellfish
Beans	green beans, baked beans, dried lentils, beans, peas, tofu, or soya bean curd
Poultry (not fried)	chicken or other poultry
Red meat and products	beef, beef burgers, pork, lamb, bacon, ham, corned beef, sausages, liver, meat pies, meat soup
Fast fried foods	fried fish in batter (as in fish and chips), fish fingers, fish cakes, chips and French fries, roast potatoes
Pastries and sweets	sweet biscuits, cakes, buns, pastries, fruit pies, tarts, crumbles, milk puddings, sponge cake, ice cream, choc ices, sweets, toffees, mints, chocolates, chocolate bars, sugar added to drinks, jam marmalade, honey, fizzy soft drinks, soda, fruit squash
Wine	wine
Olive oil	not available

**Table 2:** Characteristics of participants according to MIND diet scores at study baseline.

		MIND Diet Score			
Participant Characteristics (N = 4824)		Tertile 1 N = 1459	Tertile 2 N = 1723	Tertile 3 N = 1642	*p* ^1^
**Socio-demographics**					
Sex	(men%)	81.2	75.9	63.0	<0.001
	(women%)	18.8	24.1	37.0	
Age	(years)	60.6 ± 6.1	61.1 ± 6.0	61.3 ± 5.7	0.01
Ethnicity	(non-white%)	2.7	5.5	8.3	<0.001
	(white%)	97.3	94.5	91.7	
Marital status	(single/divorce/widow%)	25.4	21.1	23.0	0.02
	(married/cohabiting%)	74.6	78.9	77.0	
Education level	(<secondary%)	10.4	7.6	6.9	<0.001
	(secondary%)	52.0	49.8	47.3	
	(university level%)	37.6	42.6	45.8	
Socio-economic status	(low%)	9.5	8.6	8.5	0.10
	(intermediate%)	44.5	42.1	40.6	
	(high%)	46.0	49.1	50.9	
**Health behaviors**					
Smoking status	(ex/current-smokers%)	53.5	51.2	48.7	0.02
	(non-smoker%)	46.5	48.8	51.3	
Alcohol intake	(heavy%)	22.8	20.4	17.3	<0.001
	(moderate%)	60.6	65.1	69.7	
	(abstainer%)	16.6	14.5	13.0	
Physical activity	(inactive/moderately active %)	26.6	22.7	22.7	0.013
	(active%)	73.4	77.3	77.3	
Use of vitamin/mineral/food supplements ^2^	(yes%)	40.5	47.9	56.9	<0.001
Total energy intake	(kcal/d)	2240 ± 608	2163 ± 589	2063 ± 543	<0.001
**Health status**					
Body mass index	(kg/m^2^)	26.8 ± 4.2	26.6 ± 4.2	26.2 ± 4.1	<0.001
Coronary heart disease	(yes%)	8.4	8.7	8.8	0.92
Hypertension	(yes%)	36.5	38.6	38.4	0.41
Type 2 diabetes	(yes%)	8.5	9.3	9.3	0.67
Dyslipidemia	(yes%)	34.1	33.3	29.3	0.01
Cognitive deficit	(yes%)	11.1	13.8	12.9	0.07
Antecedent of depressive symptoms	(yes%)	26.2	24.4	22.4	0.05
**Outcome**					
Recurrent depressive symptoms at follow-up	(yes%)	15.1	13.5	11.4	0.01

^1^ Values are percentage or means ± standard deviation. *p*-values from analysis of variance (ANOVA) or Chi2-tests. ^2^ Information on self-reported use of vitamin/mineral and other food supplements were available in 4538 participants.

## Data Availability

Data, protocols and other metadata of the Whitehall II study are available to the scientific community either via the Whitehall II study data sharing portal (https://www.ucl.ac.uk/epidemiology-health-care/research/epidemiology-and-public-health/research/whitehall-ii). All scripts to conduct analyses are available on reasonable request from the corresponding author (T.N.A.).

## Supplementary Materials

The following supporting information can be downloaded at: https://www.mdpi.com/article/10.3390/nu16234062/s1, Table S1: Construction and Distribution of MIND scores in the 4824 Whitehall II participants, Figure S1: Study Design and Flow chart diagram mapping the selection of participants, Table S2: Comparison of included/excluded Whitehall II participants’ characteristics, Table S3: Characteristics of participants as a function of recurrence of depressive symptoms over 13 years of follow-up, Figure S2: Association between cumulative average of MIND diet score over 11-year of exposure period (1991/93–2002/04) and recurrence of depressive symptoms (DepS) over 13 years of follow-up in 4824 Whitehall II participants (3526 men and 1298 women), Table S4: Association between MIND diet score and recurrence of depressive symptoms (DepS) over 13 years of follow-up after excluding the 612 participants with cognitive impairment (N = 4212), Table S5: Association between MIND diet score and recurrence of depressive symptoms (DepS) over 13 years of follow-up after excluding the 165 participants under anti-depressive treatment prior phase 7 (N = 4659), Table S6: Association between MIND diet score and recurrence of depressive symptoms (DepS) over 13 years of follow-up after excluding the 609 participants with depression assessed through the 4-item depression subscale of the General Health Questionnaire at phase 5 (N = 4215), Table S7: Association between each MIND diet components scores (2002/04) and recurrent DepS over 13 years of follow-up in 4824 Whitehall II participants, Table S8: Association between modified MIND score * recurrence of depressive symptoms (DepS) over 13 years of follow-up; Table S9: Association between modified MIND score * recurrence of depressive symptoms (DepS) over 13 years of follow-up.
