# Supplementary material for: Adherence to MIND Diet and Risk of Recurrent Depressive Symptoms: Prospective Whitehall II Cohort Study
Source: Nutrients. 2024 Nov 26;16(23):4062. doi: 10.3390/nu16234062 (PMC11643367; doi:10.3390/nu16234062)
Supplement: Supplementary file 1 [file nutrients-16-04062-s001.zip › Supplementary tables.pdf]

Supplementary Material Tables

Supplementary Material Table S1. Construction and Distribution of MIND scores in the 4824 Whitehall II participants

**Components (servings)**	**Criteria for min. scores**	**Criteria for intermediate score**	**Criteria for maximum**	**MIND diet score in 2002/04**	**Cumulative average MIND diet score over the 11-year exposure period ***
	**0 point**	**0.5 point**	**1 point**	**Mean ± Standard Deviation**	**Mean ± Standard deviation**
Green leafy vegetables, servings	≤ 2 / week	>2 to < 6 /week	≥ 6 / week	0.78 ± 0.31	0.74 ± 0.26
Other vegetables, servings	< 5 / week	5 to 6 / week	≥ 7 / week	0.98 ± 0.12	0.98 ± 0.10
Nuts, servings	< 1 /month	1 / month to 5 /week	> 5 / week	0.33 ± 0.30	0.32 ± 0.23
Berries, servings	< 1 / week	1 / week	≥ 2 / week	0.32 ± 0.47	0.29 ± 0.35
Olive oil	Not primary oil		Primary oil	Not available in WII ± **	
Butter, Margarine (table spoon)	> 2 / day	1 to 2 / day	< 1 /day	0.60 ± 0.45	0.54 ± 0.36
Cheese, servings	≥ 7 /week	1 to 6 / week	< 1 / week	0.64 ± 0.29	0.63 ± 0.22
Whole grain, servings	<1/ day	1 to 2 /day	≥ 3 /day	0.41 ± 0.40	0.42 ± 0.32
Fish not fried, number of meals with	Rarely	1 to 3 / month	≥ 1 /week	0.79 ± 0.32	0.76 ± 0.27
Beans, number of meals with	<1 /week	1 to 3 / week	>3 / week	0.59 ± 0.39	0.57 ± 0.30
Poultry (not fried), number of meals	<1 /week	1 / week	≥ 2 / week	0.48 ± 0.50	0.43 ± 0.38
Red meat and products, number of meals	≥ 7 / week	4 to 6 / week	< 4 /week	0.51 ± 0.41	0.51 ± 0.34
Fast fried foods, number of times	≥ 4 / week	1 to 3 /week	< 1/ week	0.65± 0.32	0.63 ± 0.26
Pastries and sweets, servings	≥ 7 / week	5 to 6 / week	< 5 /week	0.17 ± 0.35	0.14 ± 0.26
Wine, one glass	> 1 / day or never	1 / month to 6 /week	1 / day	0.33 ± 0.24	0.35 ± 0.19
**Total Score (points)**	**0**	**To**	**15** (**14** in Whitehall II study)	**7.6 ± 1.6**	**7.0 ± 1.6**

MIND stands for Mediterranean-dietary approaches to stop hypertension Intervention for Neurodegenerative Delay.
For each component, the frequency of consumption of each food item portion associated with that component was summed and a concordance score of 0, 0.5, or 1 point was assigned according to dietary recommendation criteria. The total MIND score consisted of the sum of its components score (higher scores representing a healthier diet).

* When dietary exposure prior to phase 7 was available, the cumulative average of MIND diet score was calculated using the repeated measures of MIND diet score at phase 3 (1991/93, N=4994), phase 5 (1997/99, N=3798) and phase 7 (2002/200, N=4824).

**The original score includes olive oil consumption as the primary oil usually used at home, however this information was not available in Whitehall II. The overall MIND score was computed without this item.

Information on foods items included in each MIND diet component have been detailed in Table 1.

Supplementary Material Table S2. Comparison of included/excluded Whitehall II participants' characteristics

**Characteristics of participants in 2002/04**		**Participants alive in 2002/04 (n=6967)**		
		**Included (n=4824)**	**Excluded (n=2143)**	
		**% or m±SD**	**% or m±SD**	**p**
**Socio-demographic factors**				
Sex	(men%)	73.1	63.8	<0.001
Age	(years)	61.0±5.9	61.7±6.2	<0.001
Ethnicity	(non-white%)	5.6	13.7	<0.001
	(white%)	94.4	86.3	
Marital status	(single/Divorce/Widow%)	23.0	28.3	<0.001
	(married/cohabiting%)	77.0	71.7	
Socio-economic status	(low%)	8.9	17.4	<0.001
	(medium%)	42.3	45.9	
Education level	(<secondary%)	8.2	11.0	<0.001
	(secondary%)	49.6	53.5	
**Health behavior factors**				
Smoking status	(ex/current-smokers%)	48.9	46.3	0.04
Alcohol intake	(high%)	20.1	18.1	<0.001
	(moderate%)	65.3	57.5	
Physical activity	(non-active%)	23.9	32.3	<0.001
Use of vitamin/mineral/food supplements	(yes%)	48.7	49.4	0.74
Total energy intake	(kcal/d)	2152.1±584.2	2083.7±610.7	0.004
**Health status factors**				
Coronary heart disease	(yes%)	8.7	11.5	<0.001
Hypertension	(yes%)	37.9	45.4	<0.001
Type 2 diabetes	(yes%)	9.1	12.7	<0.001
Dyslipidemia	(yes%)	32.2	38.5	<0.001
Cognitive impairment	(yes%)	12.7	24.5	<0.001
Antecedent of depressive symptoms	(yes%)	24.3	27.1	<0.001
Body Mass Index	(kg/m²)	26.5±4.2	27.0±4.8	<0.001
**Exposure**				
MIND diet score 2002/04 [1]	(point)	7.6±1.6	7.5±1.6	0.23
**Outcome**				
Recurrent depressive symptoms	(yes%)	13.3	19.2	<0.001

Values are percentage or means ± standard deviation
p-values are based on X^2 test or t-test
[1] MIND diet score: higher scores representing a healthier diet.

Supplementary Material Table S3. Characteristics of participants as a function of recurrence of depressive symptoms over 13 years of follow-up

Characteristics of participants		Recurrent depressive symptoms No (n=4184)	Recurrent depressive symptoms Yes (n=640)	
		% or m±SD	% or m±SD	p
**Socio-demographic factors**				
Sex	(men%)	74.4	62.3	<0.001
Age	(years)	61.0±5.9	61.0±6.1	0.76
Ethnicity	(non-white%)	5.2	8.1	0.003
	(white%)	94.8	91.9	
Marital status	(single/divorce/widow%)	21.7	31.6	<0.001
	(married/cohabiting%)	78.3	68.4	
Socio-economic status	(low%)	8.1	14.1	<0.001
	(medium%)	41.2	49.2	
Education level	(<secondary%)	7.8	10.8	0.04
	(secondary%)	49.8	48.1	
**Health behavior factors**				
Smoking status	(ex/current-smoker%)	50.6	54.1	0.10
Alcohol intake	(high%)	20.2	19.2	<0.001
	(moderate%)	66.0	60.3	
Physical activity	(non-active%)	23.0	29.2	<0.001
Use of vitamin/mineral/food supplements[1]	(yes %)	47.9	59.4	
Total energy intake	(kcal/d)	2153±579.6	2146±613.9	0.79
**Health status factors**				
Coronary heart disease	(yes%)	8.4	10.2	0.15
Hypertension	(yes%)	37.7	38.9	0.56
Type 2 diabetes	(yes%)	8.9	10.0	0.37
Dyslipidemia	(yes%)	31.5	36.6	0.011
Cognitive impairment	(yes%)	12.0	17.0	<0.001
Antecedent of depressive symptoms	(yes%)	18.4	62.8	<0.001
Body Mass Index	(kg/m^2)	26.5±4.1	26.8±4.5	0.10
**Exposure**				
MIND diet score 2002/04 [2]	(point)	7.6±1.6	7.4±1.6	0.01

Values are percentage or means ± standard deviation
p-values are based on X^2 test or t-test

[1] Information on self-reported use of vitamin/mineral and other food supplements were available for 4538 participants.

[2] MIND diet score: higher scores representing a healthier diet.

Supplementary Material Table S4. Association between MIND diet score and recurrence of depressive symptoms (DepS) over 13 years of follow-up after excluding the 612 participants with cognitive impairment (N=4212)

	**Odds ratio (95% confidence interval) of recurrent DepS associated to tertiles of MIND diet score (531 cases/ 3681 non cases)**		
	Model 1	Model 2	Model 3
	OR (95%CI)	OR (95%CI)	OR (95%CI)
Tertile 1	Ref	Ref	Ref
Tertile 2	0.84 (0.67 to 1.05)	0.88 (0.71 to 1.10)	0.87 (0.70 to 1.08)
Tertile 3	0.65 (0.51 to 0.82)	0.70 (0.55 to 0.89)	0.70 (0.56 to 0.89)
p-linearity	*<0.001*	*0.004*	*0.02*

Logistic regression model was adjusted for sex, age, sex, total energy intake, ethnicity, marital status, socio-economic status, education level, smoking status, alcohol consumption, physical activity, coronary heart diseases, hypertension, diabetes, dyslipidemia, body mass index and antecedent of depressive symptoms.

Supplementary Material Table S5. Association between MIND diet score and recurrence of depressive symptoms (DepS) over 13 years of follow-up after excluding the 165 participants under anti-depressive treatment prior phase 7 (N=4659).

	**Odds ratio (95% confidence interval) of recurrent DepS associated to tertiles of MIND diet score (547 cases/ 4112 non cases)**		
	Model 1	Model 2	Model 3
	OR (95%CI)	OR (95%CI)	OR (95%CI)
Tertile 1	Ref	Ref	Ref
Tertile 2	0.85 (0.68 to 1.05)	0.87 (0.70 to 1.09)	0.87 (0.70 to 1.08)
Tertile 3	0.66 (0.53 to 0.84)	0.71 (0.56 to 0.89)	0.70 (0.56 to 0.89)
p-linearity	*<0.001*	*0.004*	*0.004*

Logistic regression model was adjusted for sex, age, sex, total energy intake, ethnicity, marital status, socio-economic status, education level, smoking status, alcohol consumption, physical activity, coronary heart diseases, hypertension, diabetes, dyslipidemia, body mass index and cognitive impairment.

Supplementary Material Table S6. Association between MIND diet score and recurrence of depressive symptoms (DepS) over 13 years of follow-up after excluding the 609 participants with depression assessed through the 4-item depression subscale of the General Health Questionnaire at phase 5 (N=4215)

	**Odds ratio (95% confidence interval) of recurrent DepS associated to tertiles of MIND diet score (389 cases/ 3826 non cases)**		
	Model 1	Model 2	Model 3
	OR (95%CI)	OR (95%CI)	OR (95%CI)
Tertile 1	Ref	Ref	Ref
Tertile 2	0.95 (0.74 to 1.23)	0.96 (0.74 to 1.25)	0.95 (0.74 to 1.24)
Tertile 3	0.75 (0.57 to 0.99)	0.77 (0.58 to 1.02)	0.77 (0.59 to 1.02)
p-linearity	*0.04*	*0.06*	*0.07*

Logistic regression model was adjusted for sex, age, sex, total energy intake, ethnicity, marital status, socio-economic status, education level, smoking status, alcohol consumption, physical activity, coronary heart diseases, hypertension, diabetes, dyslipidemia, body mass index and cognitive impairment.

Supplementary Material Table S7. Association between MIND diet scores (2002/04) and recurrent DepS over 13 years of follow-up after accounting for use of vitamin/mineral and other food supplements available in 4538 Whitehall II participants.

	**Odds ratio (95% confidence interval) of recurrent DepS associated to tertiles of MIND diet score**		
	In all participants N=4538[1]	In user of supplements, N=2210[2]	In non-user of supplements, N=2328[2]
	**(593 cases/ 3945 non cases)**	**(321 cases/ 1889 non cases)**	**(272 cases/ 2056 non cases)**
	OR (95%CI)	OR (95%CI)	OR (95%CI)
Tertile 1	Ref	Ref	Ref
Tertile 2	0.88 (0.70 to 1.10)	1.10 (0.79 to 1.53)	0.71 (0.51 to 0.97)
Tertile 3	0.69 (0.54 to 0.89)	0.84 (0.59 to 1.17)	0.56 (0.39 to 0.82)
p-linearity	*0.004*	*0.24*	*0.002*

[1]Logistic regression model accounted for the use of vitamin/mineral or other food supplements.

[2] Logistic regression models were stratified on use of vitamin/mineral or other food supplements.

Model was also adjusted for sex, age, sex, total energy intake, ethnicity, marital status, socio-economic status, education level, smoking status, alcohol consumption, physical activity, coronary heart diseases, hypertension, diabetes, dyslipidemia, body mass index, cognitive impairment antecedent of depressive symptoms.

Supplementary Material Table S8. Association between each MIND diet components scores (2002/04) and recurrent DepS over 13 years of follow-up in 4824 Whitehall II participants.

MIND diet Components [1] (per point)	OR	95% CI	p
Green leafy vegetable	0.55	0.42 to 0.72	<0.001
Other vegetables	0.37	0.21 to 0.66	<0.001
Nuts	0.98	0.74 to 1.30	0.89
Berries	0.72	0.59 to 0.87	<0.001
Butter, Margarine	1.17	0.95 to 1.44	0.15
Cheese	1.09	0.80 to 1.49	0.57
Whole grain	0.83	0.67 to 1.03	0.09
Fish not fried	0.70	0.54 to 0.91	0.007
Beans	0.93	0.74 to 1.15	0.49
Poultry	0.92	0.78 to 1.09	0.33
Red meat and products	1.05	0.84 to 1.30	0.68
Fast fried foods	0.74	0.56 to 0.98	0.03
Pastries and sweets	1.02	0.79 to 1.31	0.88
Wine	0.72	0.51 to 1.02	0.06

[1] For each component, the frequency of consumption of each food item portion associated with that component was summed and a concordance score of 0, 0.5, or 1 point was assigned according to dietary recommendation criteria.
For *Green leafy vegetables, Other vegetables, Berries, Nuts, Whole grain, Fish not fried, Beans and Poultry*, higher scores correspond to higher intakes.
For *Wine*, higher score corresponds to 1 glass per day, and the minimum score was attributed when participants consumed more than 1 glass/ pay or were abstainers.
For other MIND diet components *Butter/margarine, Cheese, Red meat products, Fast fried foods, Pastries and sweets* -, higher scores correspond to lower intakes of the components
Please refer to Supplementary Table 1 for additional details.

Logistic regression models were adjusted for sex, age and total energy intake.

Supplementary Material Table S9. Association between modified MIND score [1] recurrence of depressive symptoms (DepS) over 13 years of follow-up

Modified MIND score built without the following component[1] (per point)	OR	95% CI	p
Green leafy vegetable	0.95	0.89 to 1.01	0.09
Other vegetables	0.94	0.89 to 1.00	0.05
Nuts	0.95	0.89 to 1.01	0.08
Berries	0.94	0.89 to 0.99	0.04
Butter, Margarine	0.92	0.87 to 0.98	0.01
Cheese	0.94	0.88 to 0.99	0.03
Whole grain	0.94	0.88 to 1.00	0.05
Fish not fried	0.94	0.88 to 1.00	0.04
Beans	0.93	0.88 to 0.99	0.03
Poultry	0.94	0.88 to 1.00	0.05
Red meat and products	0.93	0.88 to 0.99	0.02
Fast fried foods	0.94	0.88 to 1.00	0.05
Pastries and sweets	0.93	0.88 to 0.99	0.03
Wine	0.94	0.89 to 1.00	0.05

[1]The modified MIND diet score was based on the total MIND diet score without the component i (modified MIND diet score=total MIND diet score - score of the component i).

Logistic regression model was adjusted for sex, age, sex, total energy intake, ethnicity, marital status, socio-economic status, education level, smoking status, alcohol consumption, physical activity, coronary heart diseases, hypertension, diabetes, dyslipidemia, body mass index, cognitive impairment and antecedent of depressive symptoms.
