# Supplementary material for: Adherence to MIND Diet and Risk of Recurrent Depressive Symptoms: Prospective Whitehall II Cohort Study
Source: Nutrients. 2024 Nov 26;16(23):4062. doi: 10.3390/nu16234062 (PMC11643367; doi:10.3390/nu16234062)
Supplement: Supplementary file 1 [file nutrients-16-04062-s001.zip › Supplementry figures.pptx]

## Slide 1
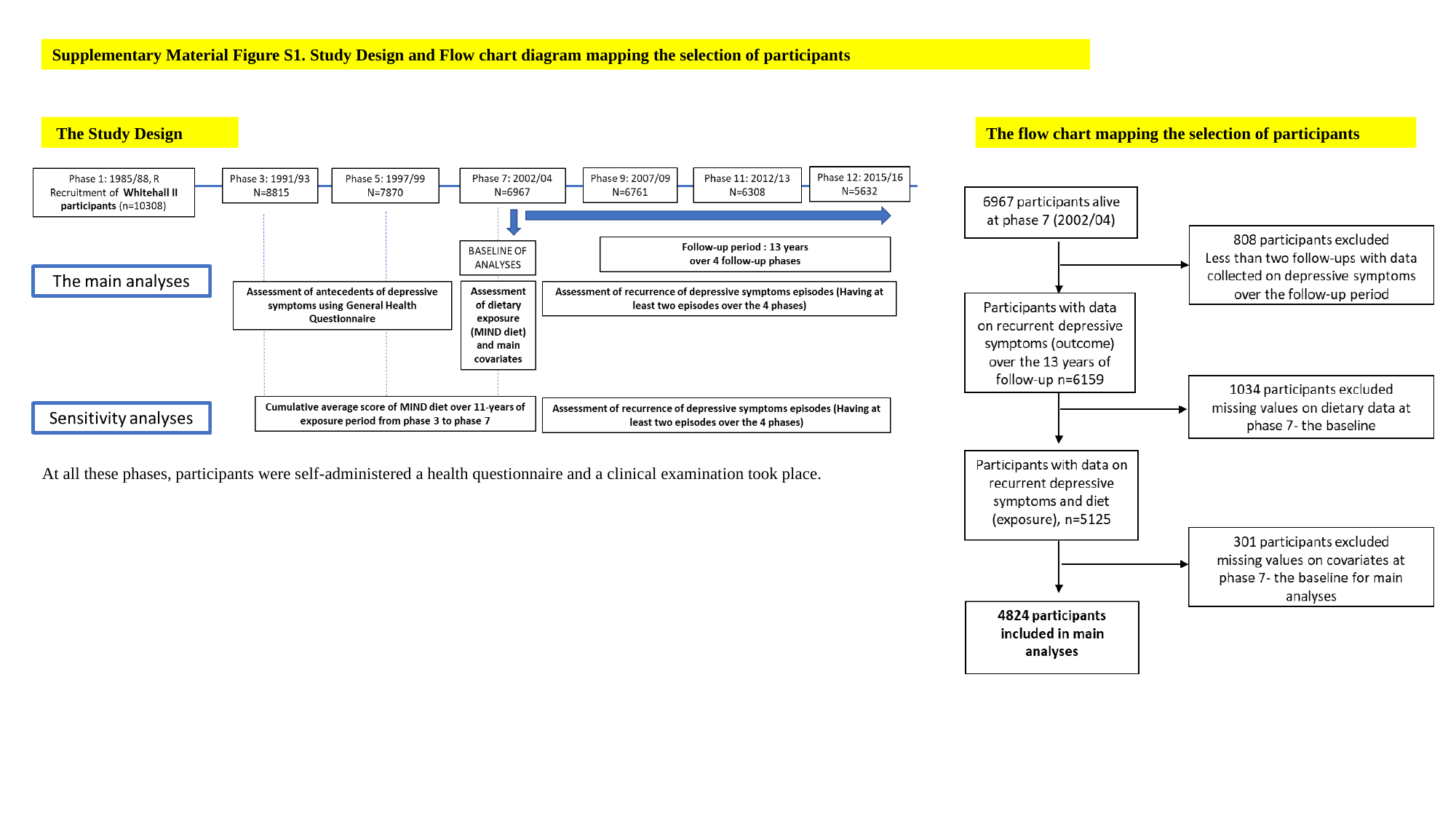

Supplementary Material Figure S1. Study Design and Flow chart diagram mapping the selection of participants
 The Study Design
The flow chart mapping the selection of participants
At all these phases, participants were self-administered a health questionnaire and a clinical examination took place.

## Slide 2
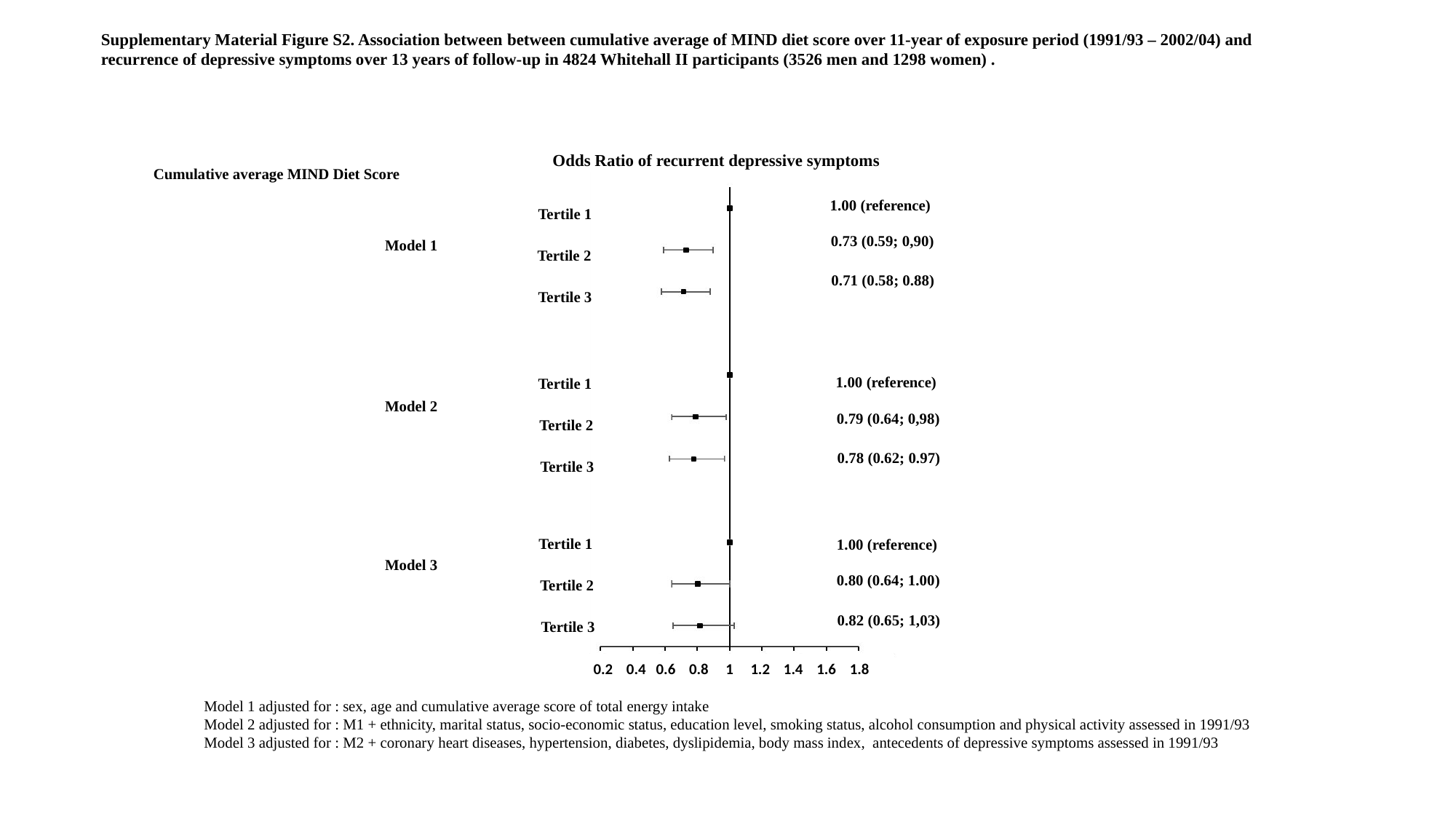

Supplementary Material Figure S2. Association between between cumulative average of MIND diet score over 11-year of exposure period (1991/93 – 2002/04) and recurrence of depressive symptoms over 13 years of follow-up in 4824 Whitehall II participants (3526 men and 1298 women) .
Odds Ratio of recurrent depressive symptoms
Cumulative average MIND Diet Score
1.00 (reference)
Tertile 1
Model 1
Tertile 2
Tertile 3
Tertile 1
Model 2
Tertile 2
Tertile 3
Tertile 1
Model 3
Tertile 2
Tertile 3
0.73 (0.59; 0,90)
0.71 (0.58; 0.88)
1.00 (reference)
0.79 (0.64; 0,98)
0.78 (0.62; 0.97)
1.00 (reference)
0.80 (0.64; 1.00)
0.82 (0.65; 1,03)
 0.2 0.4 0.6 0.8 1 1.2 1.4 1.6 1.8
Model 1 adjusted for : sex, age and cumulative average score of total energy intake
Model 2 adjusted for : M1 + ethnicity, marital status, socio-economic status, education level, smoking status, alcohol consumption and physical activity assessed in 1991/93
Model 3 adjusted for : M2 + coronary heart diseases, hypertension, diabetes, dyslipidemia, body mass index, antecedents of depressive symptoms assessed in 1991/93
